# Gene Silencing Through CRISPR Interference in Bacteria: Current Advances and Future Prospects

**DOI:** 10.3389/fmicb.2021.635227

**Published:** 2021-03-31

**Authors:** Riyu Zhang, Wensheng Xu, Shuai Shao, Qiyao Wang

**Affiliations:** ^1^State Key Laboratory of Bioreactor Engineering, East China University of Science and Technology, Shanghai, China; ^2^Laboratory of Agricultural Product Detection and Control of Spoilage Organisms and Pesticide Residue, Faculty of Food Science and Engineering, Beijing University of Agriculture, Beijing, China; ^3^Shanghai Collaborative Innovation Center for Biomanufacturing Technology, Shanghai, China; ^4^Shanghai Engineering Research Center of Maricultured Animal Vaccines, Shanghai, China

**Keywords:** CRISPR/Cas, gene silencing, essential genes, Tn-seq, CRISPRi screen

## Abstract

Functional genetic screening is an important method that has been widely used to explore the biological processes and functional annotation of genetic elements. CRISPR/Cas (Clustered regularly interspaced short palindromic repeat sequences/CRISPR-associated protein) is the newest tool in the geneticist’s toolbox, allowing researchers to edit a genome with unprecedented ease, accuracy, and high-throughput. Most recently, CRISPR interference (CRISPRi) has been developed as an emerging technology that exploits the catalytically inactive Cas9 (dCas9) and single-guide RNA (sgRNA) to repress sequence-specific genes. In this review, we summarized the characteristics of the CRISPRi system, such as programmable, highly efficient, and specific. Moreover, we demonstrated its applications in functional genetic screening and highlighted its potential to dissect the underlying mechanism of pathogenesis. The recent development of the CRISPRi system will provide a high-throughput, practical, and efficient tool for the discovery of functionally important genes in bacteria.

## Introduction

Genome editing is a robust technology of modifying genome with a high efficiency emerging in recent years, which has a growing and profound influence on bioscience, biotechnology, and bio-industry. ZFN and TALEN, the primary generation genome editing technologies, are protein-guided and need protein engineering, which is time-consuming and not easy to operate (Kim et al., 1996; Cathomen and Joung, 2008; Wood et al., 2011). CRISPR/Cas-based genome editing hereby came into being and could theoretically edit the genome of any organism. So far, the powerful technology has brought about a revolution in biology due to its significantly simplified construction process.

In general, essential genes are hard to be probed because their knock-out is lethal to the organism. Gene silencing technology such as RNA interference (RNAi) is capable of inhibiting the expression of genes and, hence, is applied to investigate the function of essential genes (Agrawal et al., 2003). Since RNAi is mainly utilized in eukaryotes, a silencing tool is also required for dissecting the essential genes in the prokaryote system. CRISPRi has been developed from the CRISPR/Cas-based genome editing to fill in the blanks. Furthermore, coupling with high-throughput sequencing, it has emerged as a potential and promising strategy to perform functional genomics research in bacteria. Here, this review gives a brief introduction to the CRISPRi system, the underlying mechanism and properties, and highlights its application as a high-throughput screening tool in gene functional analysis.

## The Mechanism of the CRISPR/Cas-Based Genome Editing and Interference System

CRISPR/Cas-based genome editing is a newly developed RNA-guided genome editing system. CRISPR is a series of clustered DNA sequences including repeats and spacers and Cas are CRISPR-associated proteins (Jansen et al., 2002). The CRISPRs are observed in nearly 90% of genomes of the sequenced archaea and nearly 40% of genomes of the sequenced bacteria (Sorek et al., 2008). They can be divided into two classes based on the number of Cas proteins interfering with an invading DNA (Makarova et al., 2015). Class 1 systems include type I, III, and IV, which have multi-subunit effector complexes and, hence, are not suitable to be applied to genome editing. Instead, Class 2 systems consist of type II, V, and VI, which only possess a single effector protein. Moreover, type II is the simplest CRISPR/Cas systems and can achieve interference with an invading DNA only by a single multi-functional effector Cas protein (Makarova et al., 2011).

As the adaptive immune systems of prokaryote, CRISPR/Cas systems can recognize and cleave foreign nucleic acids (Barrangou et al., 2007; Brouns et al., 2008). With the help of a chimeric single guide RNA (sgRNA), the Cas protein is targeted to a specific DNA sequence and then triggers a double-strand break (DSB) at the chromosomal DNA (Deltcheva et al., 2011). The recognized short DNA sequence is called the protospacer adjacent motif (PAM) and different Cas proteins can recognize different PAM sequences. For instance, the recognized PAM of SpCas9 protein derived from *Streptococcus pyogenes* is NGG whereas the recognized PAM of StCas9 from *Streptococcus thermophilus* is NGGNG (Cho et al., 2013; Karvelis et al., 2013). Coupled with an available and editing template DNA, such DSBs could be repaired through homologous recombination (HR) to introduce precise genome editing. Instead, such DSBs could also be repaired by Non-homologous end joining (NHEJ), which would produce small insertion and deletion mutations to abolish or disrupt the function of the target gene.

**FIGURE 1:**
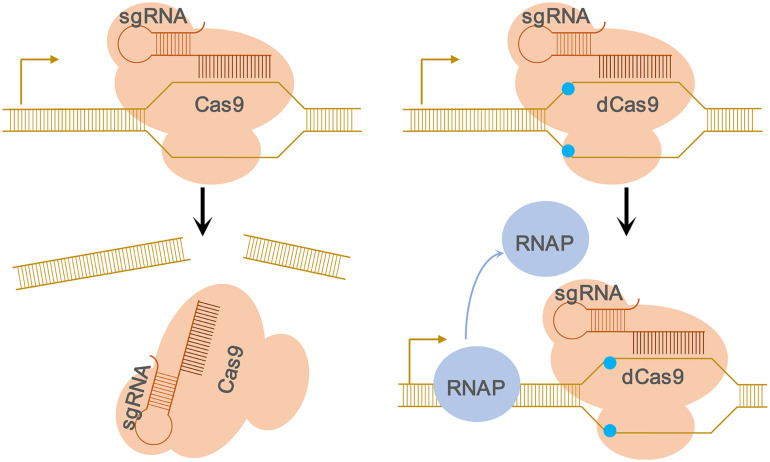
Schematic of the CRISPRi approach. **Left**, Cartoon representation of the CRISPR-mediated regulation of gene expression. The wild-type Cas9 protein binds to the sgRNA and forms a protein–RNA complex. Once Cas9–sgRNA complex binds to specific DNA target adjacent to PAM, it leads to the cleavage of the target DNA due to the nuclease activity of the Cas9 protein. **Right**, Cartoon depicting the CRISPRi-mediated interference of gene expression caused by nuclease-deficient dCas9. The nuclease-deficient dCas9 contains two substitutions in the nuclease domains (D10A and H840A, blue dots), and thus lose the endonuclease activity. If the target DNA sequence locates inside an open reading frame, the dCas9–sgRNA–DNA complex will block the movement of RNAP and subsequent transcription elongation, resulting in transcription inhibition of the target gene.

As one of the most commonly used Cas proteins, the Cas9 protein can cleavage the invading DNA because it possesses RuvC and HNH nuclease domains which can cleave the non-complementary strand and the complementary strand, respectively (Jinek et al., 2012). The catalytic domains of Cas9 are mutated to generate the inactive dCas9 (nuclease – dead mutants of Cas9) lacking the endonuclease activity but instead, it still can be in conjunction with the sgRNA (Jinek et al., 2012). Consequently, the dCas9-sgRNA complex specifically binds to the target gene at the promoter or coding sequence and acts as a roadblock to the elongating RNA polymerase, hence, aborting transcription initiation, or elongation. The function of dCas9 was confirmed by the native elongating transcript sequencing (NET-seq) experiment (Qi et al., 2013) and Figure 1 demonstrated the RNAP is blocked by the dCas9-sgRNA complexes.

To achieve a CRISPR/Cas-based interference, scientists have already developed multiple strategies such as the plasmid-based system and the chromosomally integrated system. The plasmid-based system consists of the single-plasmid system and the dual-plasmid system. The single-plasmid system employs a composite plasmid harboring dCas9 and sgRNA together, while the major limitation is the cloning efficiency due to the relatively large plasmid size. The construction of the dual-plasmid system is simplified, in which dCas9 and sgRNA are carried by two independent small plasmids. However, the plasmid incompatibility and stability have to be taken into consideration before applying it. Both of the plasmid-based systems have no need to integrate elements into the genome of bacteria, avoiding the unexpected consequences of the change of genome, and have been extensively applied to silence single gene or multiple genes in bacteria (see section “Application of CRISPRi in Bacteria”). Alternatively, the chromosomally integrated system was developed and the dCas9 is integrated into a neutral site of the bacterial genome. The sgRNA exists in a small plasmid similar to that of the plasmid-based system and the establishment of sgRNA assessment algorithm enables the design of a high-saturated sgRNA plasmid library. With the decreasing cost of DNA synthesis, it is feasible to synthesize large sgRNA libraries, leveraging the chromosomally integrated system for almost all the high-throughput CRISPRi screening in bacteria (see section “Application of CRISPRi in High-Throughput Screen”).

## Properties of CRISPRI

The CRISPRi technique is originated from CRISPR and, thus, possesses many properties as same as what CRISPR owned. They are both programmable, highly efficient, and specific but also face the troubles of off-target and toxicity. What is more, there are some distinct properties from the CRISPR/Cas-based genome editing including both the merits and demerits (Table 1).

**TABLE 1:** The advantages and disadvantages of CRISPRi.

Advantages	Disadvantages
Tunable knockdown	Off-target
Inducible knockdown	Bad-seed effect
Reversible knockdown	Polar effect and reverse polar effect
Controlling multiple genes	Toxicity of dCas9

Many advantages emerge in CRISPRi. First of all, CRISPRi could simultaneously regulate the expression of multiple genes, expanding the breadth of this application. Qi et al. (2013) found that CRISPRi could be applied to regulate multiple genes independently without crosstalk. Kim et al. (2017) used the tunable CRISPRi system to repress the expression of multiple genes and achieved the increment of n-butanol yield and productivity in recombinant *Escherichia coli*. Lv et al. (2015) utilized CRISPRi to manipulate the expression of multiple essential genes involved in 4HB synthesis and then regulate P(3HB-co-4HB) composition.

Not only the expression of genes, but also the degree of gene repression could be controlled. Tuning gene repression is helpful because some genes are extremely sensitive to knockdown and many genes of interest are expected to be expressed under tight control. This can be fulfilled by titrating the concentration of dCas9 or sgRNA from an inducible promoter, which is easy and straightforward. Li et al. (2016) modulated the expression levels of target genes via controlling the expression of dCas9 under the control of P*_BAD_* promoter, resulting in over two orders of magnitude dynamic range. Fontana et al. (2018) achieved a broad range of titration of the CRISPRi repression by changing the level of gRNA from the P*_tet_* promoter in *E. coli*. Partial repression of genes could also be realized by introducing mismatches between the target and gRNA. The method is suitable when the bacteria is sensitive to the level of dCas9 required for maintaining at a low concentration. Bikard et al. (2013) took advantage of the mismatches between the crRNA and target DNAs to modulate their repression level in *E. coli*.

In addition, CRISPRi-based knockdown is inducible and reversible, which enables the temporal and dynamic regulation of interested genes. When dCas9 is under the tight control of the anhydrotetracycline-inducible (aTc-inducible) promoter, the knockdown could be either be induced by aTc or reversed by removing the inducer from the culture (Qi et al., 2013). Likewise, the arabinose could activate the expression of dCas9 from the P*_BAD_* promoter to induce a CRISPRi-based knockdown and this silencing is reversible once the inducer is washed away from the media (Li et al., 2016). Wang T. et al. (2019) found that genes of *Yersinia pestis* silenced by CRISPRi might restore expression by washing away the inducer in an actively replicating bacteria.

On the other hand, there are many disadvantages in CRISPRi including the bad-seed effect, polar effect and reverse polar effect, toxicity, and off-target. Bad-seed effect was defined as sgRNAs with specific 5-nt seed sequences that can produce strong fitness defects regardless of the other 15-nt of the guide sequence (Cui et al., 2018). Similarly, polar effect and reverse polar effect could disturb the result of the CRISPRi screen. Peters et al. (2016) reported the polar effect when sgRNA blocking a gene will repress the expression of all downstream genes in an operon. It is expected because once blocked by dCas9, RNAP is hard to go forward and trigger the transcription of downstream genes (Peters et al., 2016). In addition, when the dCas9-sgRNA complexes were guided to non-essential genes located upstream of the essential genes in operons, it exhibited a strong impairment on cell fitness (Cui et al., 2018). It is not surprising that disrupting the transcription of an upstream gene will cause a depletion of the cotranscribed downstream genes as well because they are often carried on a single transcript. Intriguingly, the reverse polar effect was also observed when the dCas9-sgRNA complexes might silence the upstream of targeted genes (Peters et al., 2016; Cui et al., 2018). The reverse polar effect could be explained due to the destabilization of the interrupted transcript.

Like Cas9, the degree of toxicity of dCas9 has been demonstrated in many bacteria. Lee et al. (2019) found that dCas9 can lead to a longer lag phase of *Vibrio natriegens*, which indicated the marginal toxicity of dCas9. The high-level dCas9 severely decreased the growth rate of *E. coli* and changed the cell shape to an abnormal filamentous morphology (Cho et al., 2018). High-level dCas9 up-regulated the genes associated with cell division and down-regulated the genes encoding proteins located in the cell membrane. The dCas9 directly bound upstream of 37 genes without sgRNA including *fimA* encoding bacterial fimbriae. A high concentration of dCas9_Spy_ was lethal to *Mycobacterium tuberculosis* without a target sgRNA (Rock et al., 2017). Instead, Zhang and Voigt constructed a non-toxic version of dCas9 (dCas9^∗^_PhlF) to avoid off-target effects, that binds to DNA through PhlF instead of dCas9 (Zhang and Voigt, 2018). Hence, it is worth noting that the effect on the growth of recipient strains by dCas9 should be tested before applying CRISPRi.

Furthermore, off-target effects appeared in CRISRPi. The sgRNAs with 9-nt of identity in the seed sequence can produce off-target effects in *E. coli* MG1655 (Cui et al., 2018). In general, off-targeting is rarely encountered in bacteria, in part because the relatively small genome size of bacteria limits the potential for sites with only one or two mismatches similar to the target sequence (Bikard et al., 2013; Rock et al., 2017). Indeed, we could not rule out the possibility of off-target effects, hence, target sites should be cautiously determined to ensure that they are unique and not highly similar to other sites in the genome.

## Comparison Between the CRISPRI and sRNA-Mediated Gene Silencing

Before the emergence of CRISPRi, the gene silencing by RNA interference (RNAi) with small RNA (sRNA), a series of RNA whose length is within 50–200 nt such as miRNA and siRNA, have been developed as a powerful tool of gene silencing in eukaryotes. As shown in Table 2, there are many common characteristics between the sRNA-mediated gene silencing and CRISPRi technique including programmable, off-target, simultaneous inhibition of the expression of multiple genes, etc. However, the CRISPRi technique has its distinct properties. First, the inhibition of the initiation and elongation of RNAP by the dCas9-sgRNA complex is at a transcription level, while the block of the initiation of the ribosome by sRNA is at a post-transcription level. Second, the objects and sites of the target are different. The target of sRNA-mediated gene silencing is commonly the 5′ UTR of mRNA while CRISPRi will target the promoter or the ORF of interesting genes (Na et al., 2013), which enables a more stable and efficient interference. Lastly, the sRNA-mediated gene silencing mainly needs the help of other chaperon proteins. For instance, the RNA chaperon Hfq is required for base pairing and thus stabilize the interaction between sRNA and its target mRNA (Beisel and Storz, 2010). Whereas, CRISPRi functions via dCas9 protein acting as a roadblock (Man et al., 2011). Therefore, CRISPRi is a more robust and widely used tool in gene silencing.

**TABLE 2:** Comparison of sRNA-mediated gene silencing and CRISPRi.

Characteristic	sRNA-mediated gene silencing	CRISPR interference
Silence type	post-transcriptional inhibition	transcription inhibition
Target	5′ UTR of mRNA	promoter or the ORF of interested genes
Required tools	sRNA and chaperone protein (Hfq, etc.)	sgRNA and dCas9
Common characteristics	silencing through base pairing of RNA and target, silence simultaneously multiple genes, programmable, off-target	

## Comparison Between CRISPRI Screen and Tn-seq

Genome-wide screening could associate genes with phenotypes at a large scale in bacteria. Transposon sequencing was applied extensively in functional genomics research (van Opijnen et al., 2009). A saturated transposon insertion library is cultured in competitive conditions and the fitness for each mutant can be determined through NGS analysis. However, there are some certain technical limitations that it is impossible to investigate essential genes because the transposons inserted strains might exhibit growth deficiency and that not all Tn-insertions result in gene inactivation. Accordingly, the Tn-seq screen requires large libraries to fully cover the genome and eventually (Yang et al., 2017), this will lead to a large amount of nonsense mutations. The complexity of mutant libraries may cause a bottleneck effect during the following screens.

CRISPR interference screen could facilitate functional analysis of essential genes because it can probe both non-essential and essential genes. Moreover, the CRISPRi screen is not only applicable to genome-wide but also a tiling library for specific genes of interest, which is cost-saving. The genome-wide library or tiling library could be selected when designing sgRNA. Hence, the CRISPRi screen library is more flexible than the Tn-seq due to its adjustable library size (Table 3). In addition, the CRISPRi screen is more suitable for mapping phenotypes to short genes than the Tn-seq as the latter may produce poor statistical robustness when short genes such as non-coding RNAs are investigated (Wang et al., 2018). However, the off-target effects have hindered the application of the CRISPRi screen. In this view, the Tn-seq has an obvious advantage over the CRISPRi screen in terms of the accuracy, therefore, the combination of the CRISPRi screen and Tn-seq will provide a reasonable and effective strategy in functional genomics research.

**TABLE 3:** Comparison of Tn-seq and CRISPRi screen.

Characteristic	Tn-seq	CRISPRi screen
Mutation type	insertion mutation	knock-down
Required tools	transposon	sgRNA, dCas9
Gene selection	random insertion	random design or specific design
Gene type	non-essential gene	non-essential gene or essential gene
Library size	genome-wide library	genome-wide library or tiling library
Advantages	suitable for operon genes	adjustable library size, suitable for essential gene, and short genes
Disadvantages	unsuitable for essential gene and short genes, transposon insertion prefers TA site, which results in insertion sites uneven distribution.	off-target, polar and reverse polar effect, bad-seed effect, dCas9-specific toxicity

## Application of CRISPRI in Bacteria

CRISPR interference could be applied to perform a functional analysis of specific genes in pathogens. Scientists established a CRISPRi-based knockdown system to achieve the attenuation of virulence in the animal model when virulence genes were silenced, which lay the foundation for probing the virulence-associated essential genes of the unknown function in pathogens. The Mobile-CRISPRi system was established including modular and transferable components that can be integrated into the genomes of diverse bacteria to expand the range of the CRISPRi systems within bacteria (Peters et al., 2019). Mobile-CRISPRi was used to control the expression of conditionally essential (CE) virulence genes in a murine model of pneumonia with the purpose of dissecting the function of CE genes. Based on this analysis, the gene *exsA*, a CE gene encoding the type III secretion system activator, was identified to repress and inhibit the secretion of effectors and attenuate virulence in mice (Qu et al., 2019). Wang T. et al. (2019) introduced an optimized CRISPRi system into *Yersinia pestis* and thereby repressed virulence-associated genes *yscB* or *ail*, resulting in the virulence attenuation in HeLa cells and mice, in line with the previously reported phenotypes caused by *yscB* and *ail* knockout.

By introducing CRISPRi into the bacterial pathogen, the essential genes intimately tied to viability can be characterized, which provided several novel targets for vaccine and antibiotic development. Caro et al. (2019) focused on essential genes involved in viability and virulence in *Vibrio cholerae* and identified that the reduced expression of the lipoprotein transport (Lol) system rendered cells prone to plasmolysis and resulted in dynamic membrane rearrangements and extrusion of mega outer membrane vesicles, which thus provided a novel drug target. In *Streptococcus pneumoniae* serotype 2 strain D39, the genes *murT* and *gatD* were determined as the essential genes for peptidoglycan synthesis (Liu et al., 2017). Also, *tarP* and *tarQ* involving in the polymerization of teichoic acid precursors were also identified (Liu et al., 2017), which would contribute to the development of novel vaccines and antibiotics.

Besides, CRISPRi could be applied to the biosynthesis of commodity chemicals. Scientists utilized CRISPRi to increase the production of chemicals in bacteria. CRISPRi was used to silence the genes on the branch pathways of surfactin synthesis and thereby enhance the amino acid precursor supply in order to increase the production of surfactin in *Bacillus subtilis* (Wang C. et al., 2019). Improved production of anthocyanin peonidin 3-O-glucoside (P3G) was realized through repressing the transcriptional repressor MetJ to downregulate the methionine biosynthetic pathway in *E. coli* (Cress et al., 2017). In *Corynebacterium glutamicum*, the shikimic acid yield was increased by altering the expression of related genes (Zhang et al., 2016).

Apart from the biosynthesis of natural chemicals, the chemical composition can be modified by CRISPRi. For example, polyhydroxyalkanoates (PHA), a family of biodegradable and biocompatible polyesters consisting of poly (3-hydroxybutyrate-co-4-hydroxybutyrate) [P(3HB-co-4HB)], possesses similar properties with traditional plastics with its physical nature depending on the ratio of 3HB and 4HB. In order to meet the demand of diverse industrial applications, the expression of multiple essential genes involved in 4HB synthesis was manipulated resulting in the modification of P(3HB-co-4HB) composition (Lv et al., 2015). As another example, the recombinant *E. coli* harboring *phaCAB* operon was widely applied to produce Polyhydroxybutyrate (PHB). The activity of PhaC is direct to PHB accumulation but reverse to PHB molecular weight. Li et al. (2017) precisely controlled the expression of *phaC* by targeting diverse sites by sgRNA with the aim of modulating the balance between PHB accumulation and PHB molecular weight.

## Application of CRISPRI in High-Throughput Screen

Combined with high-throughput screen work, CRISPRi has been performed extensively to investigate the phenotypes of essential genes involved mainly in cell morphology and growth in prokaryotes. Peters et al. (2016) used the CRISPRi screening to discover essential genes intimately tied to cell morphology in the Gram-positive model bacterium *Bacillus subtilis*. Lee et al. (2019) identified a minimal set of genes required for the rapid growth of the fast-growing bacterium *Vibrio natriegens* contributing to further research and engineering of *Vibrio natriegens*. Rousset et al. (2018) identified *E. coli* genes required by phages λ, T4, and 186 for the production of functional progeny, which thus provided novel insights into the design of improved phage therapies. A thorough CRISPRi screening in *Synechocystis* sp. PCC 6803 lead to the identification of *gltA* and *pcnB* facilitating the productivity of L-lactate and *bcp2*, the L-lactate tolerance related gene (Yao et al., 2020). A pooled CRISPRi screen facilitated the discovery of growth switches *sibB*/*ibsB*, which can be applied to decoupling cell growth and protein production in *E. coli* (Li et al., 2020).

Furthermore, the CRISPRi screen could be used to reveal the properties and design rules of itself. Coupling the CRISPRi screen with machine learning, the bad-seed effect was found and furthermore, it could be alleviated by the reduced dCas9 concentration (Cui et al., 2018). Wang et al. (2018) reported that sgRNAs targeting to the first 5% of the ORF near the start codon increased the efficiency of silencing in *E. coli* MG1655 and they also defined 10 sgRNAs/gene as the minimal sufficient number for reliable hit-gene calling. Calvo-Villamanan et al. (2020) provided a novel model to predict on-target activity for dCas9 based on the target sequence in *E. coli*, especially in bases surrounding the PAM sequence when dCas9 binds to the coding strand. The result indicated that the silencing activity of dCas9 was not only determined by sgRNA but also by the target sequence (Calvo-Villamanan et al., 2020).

## Future Perspectives

Over the last few years, high-throughput CRISPRi screens have been performed with a veritable explosion of high-throughput, high-dimensional of essential genes, and conditional essential genes. These studies have revealed numerous new biology, not only novel gene functions but also novel connections within gene networks.

As a promising tool in bacterial genome engineering, CRISPRi screens have become increasingly common in diverse bacteria. However, the main challenge for CRISPRi application in bacteria is the off-target effect. To overcome this hurdle, the engineering of dCas9 variants is on-going. Recently, an expanded PAM SpCas9 variant (xCas9) was established and has a much greater DNA specificity than most commonly used SpCas9, resulting in substantially lower off-target effects at genome-wide targeted sites (Hu et al., 2018). On the other hand, optimizing sgRNA design is thought to be a useful strategy to minimize off-target effects. In *E. coli*, a high-density and comprehensive sgRNA on-target activity map was constructed and then used to guide the optimization of sgRNA on-target activity prediction algorithm, aiming to accurately predict highly effective sgRNAs (Guo et al., 2018).

**FIGURE 2:**
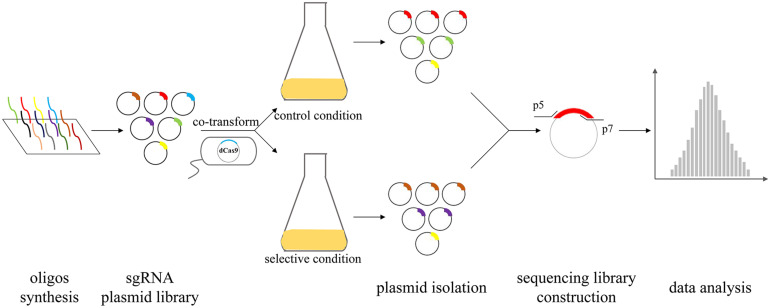
The CRISPRi screening procedure in bacteria. Firstly, oligos designed on a large scale by the software are synthesized through a chip and then, the sgRNA plasmids library is constructed. Mix sgRNA plasmids are co-transformed into recipient strains in which dCas9 is integrated into genomic DNA. Competitive growth culture is performed in selective condition and control conditions. The mixed plasmids are isolated from pooled colonized strain and subsequently used for high-throughput sequencing. Finally, data analysis of the relative fitness contribution of each gene is performed.

A powerful tool for silencing genes is required for dissecting the underlying mechanism of pathogenesis. Compared to transposon-based approaches, CRISPRi libraries are more compact, which can enable proper use in situations where Tn-seq would be bottlenecked with similar genomic coverage. The sgRNA plasmid design, which contains the 20-bp target sequence, facilitates an easy and efficient cloning that is readily scalable for the construction of sgRNA libraries for genome-wide gene targeting. A concise pooled CRISPR interference system was recently built for high-throughput quantitative genetic interaction screening on a genome-wide scale for the important human pathogen *S. pneumoniae* (Liu et al., 2021). Several *S. pneumoniae* genes were identified as essential in a laboratory medium whereas exhibited neutral in the host. This gives us a hint that the role of essential genes of pathogens might be overlooked during the infection due to conventional mutagenesis. Based on this idea, high-throughput *in vivo* evaluation of the fitness cost of genes by CRISPRi screen can broaden our horizons of pathogenesis research (Figure 2). CRISPRi screen can be used to probe essential genes that are difficultly characterized by Tn-seq, thus, is a complement to Tn-seq in high-throughput methods to facilitate the mapping of genotype-phenotype associations of core and more essential genes. The CRISPRi screen has the prospect of becoming a powerful tool screening vaccine and drug targets because many essential genes that are potential targets previously rarely researched. The knowledge of conditional essential genes acquired from CRISPRi screen will provide new insights and expand our current understanding of the functional genomics in prokaryotes.

## Author Contributions

SS and QW designed the mini review. All authors wrote, revised, and approved the manuscript.

## Conflict of Interest

The authors declare that the research was conducted in the absence of any commercial or financial relationships that could be construed as a potential conflict of interest.
